# Complete nucleotide sequences and annotations of *φ*673 and *φ*674, two newly characterised lytic phages of *Corynebacterium glutamicum* ATCC 13032

**DOI:** 10.1007/s00705-018-3867-x

**Published:** 2018-05-15

**Authors:** Yurgis A. V. Yomantas, Elena G. Abalakina, Juliya S. Lobanova, Victor A. Mamontov, Nataliya V. Stoynova, Sergey V. Mashko

**Affiliations:** Ajinomoto-Genetika Research Institute, 1st Dorozhny pr. 1-1, 117545 Moscow, Russian Federation

## Abstract

The genomes of two new lytic phages of *Corynebacterium glutamicum* ATCC 13032, *φ*673 and *φ*674, were sequenced and annotated (GenBank: MG324353, MG324354). Electron microscopy studies of both virions revealed that taxonomically they belong to the *Siphoviridae* family and have a polyhedral head with a width of 50 nm and a non-contractile tail with a length of 250 nm. The genomes of *φ*673 and *φ*674 consist of linear double-stranded DNA molecules with lengths of 44,530 bp (G+C = 51.1%) and 43,193 bp (G+C = 50.7%) and identical, protruding, cohesive 3’ ends 13 nt in length. The level of identity between the *φ*673 and *φ*674 genomes is 85.2%. Two major structural proteins of each virion were separated via SDS-PAGE and identified using peptide mass fingerprinting. Based on bioinformatic analysis, 56 and 54 ORFs were predicted for *φ*673 and *φ*674, respectively. Only 20 of the putative gene products of *φ*673 and 20 of *φ*674 could be assigned to known functions. Both genomes were divided into functional modules. Nine putative promoters in the *φ*673 genome and eight in the *φ*674 genome were predicted. One bidirectional Rho-independent transcription terminator was identified and experimentally confirmed in each phage genome.

**Electronic supplementary material:**

The online version of this article (10.1007/s00705-018-3867-x) contains supplementary material, which is available to authorized users.

## Introduction

*Corynebacterium glutamicum* is a nonpathogenic, gram-positive bacterium that is widely used for the industrial production of a broad range of substances, including amino acids and proteins [1]. In many cases, phages are responsible for the lysis of commercially interesting strains during fermentation, which leads to financial losses in the biotechnology industry. Many corynephages have been isolated, but only a few of them have been completely sequenced [2–4]. In the present study, the genomes of *φ*673 and *φ*674, two newly identified lytic phages of *C. glutamicum* ATCC 13032, were sequenced and annotated. The phages *φ*673 and *φ*674 were obtained from VKPM (the Russian National Collection of Industrial Microorganisms at the Institute of Genetics and Selection of Industrial Microorganisms, Moscow). Four genes associated with sensitivity to *φ*674 were identified in the *C. glutamicum* ATCC 13032 genome and could be useful for the construction of phage-resistant strains [5]. The newly constructed cosmid based on *cos*-sites of *φ*674 could be helpful for improving genetic tools for *C. glutamicum*, particularly with respect to the non-specific transduction of DNA fragments between *C. glutamicum* ATCC 13032 strains; such transduction has been reported for other phage-host systems [6, 7].

## Results and discussion

Phages *φ*673 and *φ*674 were propagated on *C. glutamicum* ATCC 13032 and purified via centrifugation in a CsCl gradient as previously described [8].

**Fig. 1:**
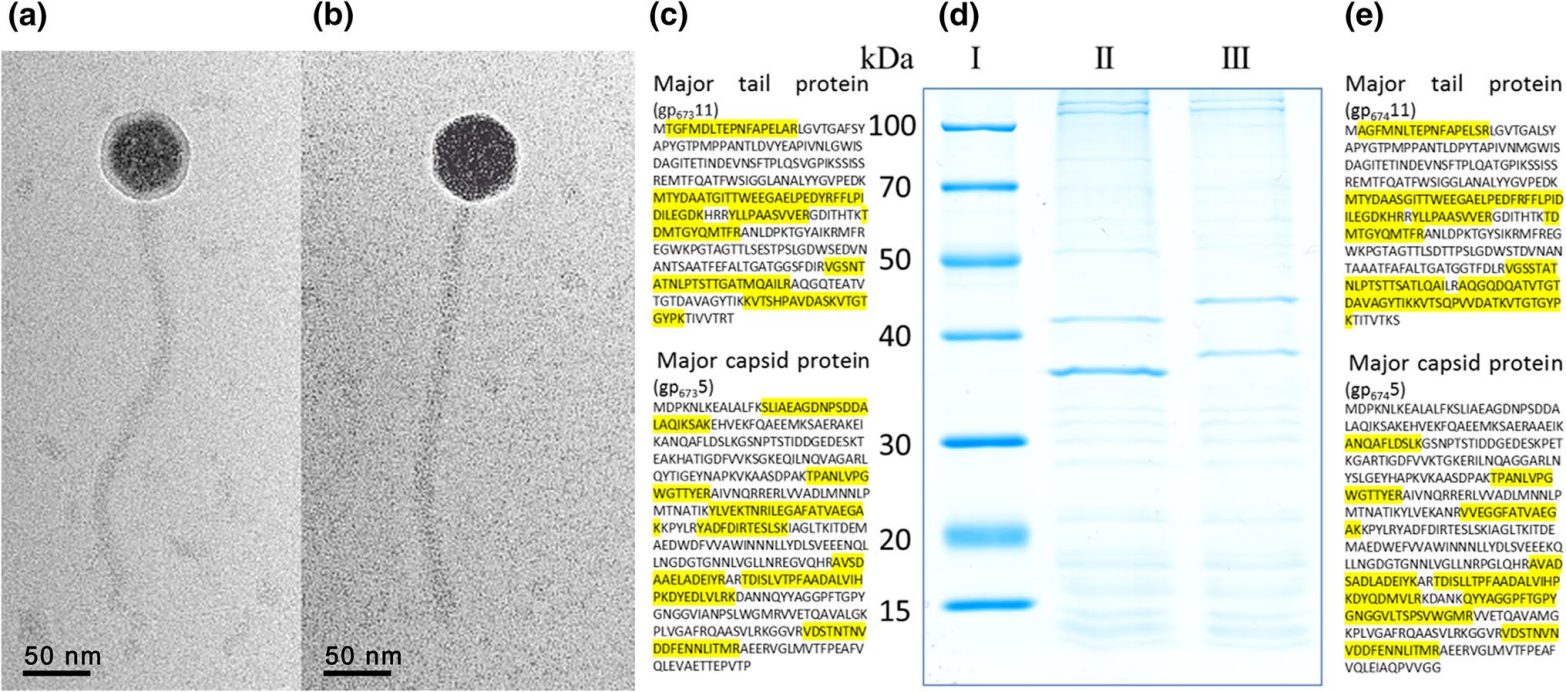
(a, b) Electron micrograph of *φ*673 and *φ*674 phages. Bar, 50 nm. (d) SDS-PAGE analysis of *φ*673 and *φ*674 structural proteins. Molecular weight markers (lane I). Protein profile of *φ*673 (lane II), *φ*674 (lane III). (d) Two major proteins bands from the *φ*673 and *φ*674 phages underwent peptide mass fingerprinting analysis; (c, f) the corresponding predicted amino acid sequences (not highlighted) and the aa sequences detected in the analysis (highlighted) are shown

Transmission electron microscopy studies of these two phages revealed that their virions belong to the *Siphoviridae* family. Both virions had a polyhedral head with a width of 50 nm and a long non-contractile tail with a length of 250 nm and a diameter of 11 nm (Fig. 1a, b). The putative gene products (gp) gp_*φ*673_14 and gp_*φ*674_14 were assigned to the tail tape measure protein (TMP). For both phages, the relationship between the observed tail length (~ 250 nm) and TMP size (1,577 aa for *φ*673 and 1,572 aa for *φ*674), which involved a ratio of 0.159 nm/aa, was reasonable [9].

Purified genomic DNA from both phages was sequenced using Illumina technology at Evrogen (Moscow, Russia, http://www.evrogen.ru). Sequences of *cos*-sites were determined in run-off experiments and were compared with the nucleotide sequences of the ligated phage ends.

Two online bioinformatic programs, Glimmer3 (https://www.ncbi.nlm.nih.gov/genomes/MICROBES/glimmer_3.cgi) and GeneMark S (http://exon.biology.gatech.edu/), were used to search for ORFs. InterPro (http://www.ebi.ac.uk/interpro/) was used to improve the initial annotation of predicted proteins. Putative promoters were searched using phiSITE’s PromoterHunter (with parameters for “-10” and “-35” [Supplementary Fig. 1]) (http://www.phisite.org/main/index.php?nav=tools&nav_sel=hunter). Bi-directional, while rho-independent transcription terminators were identified using ARNold: finding terminators (http://rna.igmors.u-psud.fr/toolbox/arnold/index.php).

The *φ*673 and *φ*674 genomes consist of linear double-stranded DNA molecules with lengths of 44,530 bp (G+C = 51.1%) and 43,193 bp (G+C = 50.7%), respectively, and share identical, protruding, cohesive 3’ ends 13 nt in length (AGAAGGGGGCGGA-3’). A cosmid vector for molecular cloning has been constructed on the basis of the phage *φ*674 *cos*-site, and the functionality of the *cos*-site was experimentally confirmed (unpublished results). Based on bioinformatics analysis, 56 and 54 ORFs were identified in the *φ*673 and *φ*674 genomes, respectively. These ORFs cover approximately 97% and 96% of the entire *φ*673 and *φ*674 genomes, respectively. Only 20 gene products (gps) from each phage could be assigned to known biological functions (Supplementary Table 1, 2); the other 17 and 16 gp(s) exhibiting homology to hypothetical proteins, while 19 and 18 ORFs present in *φ*673 and *φ*674, respectively, had no homologues in the databases. No tRNA genes were identified in either phage genomes.

Nine and eight putative promoters were predicted in the *φ*673 and *φ*674 genomes, respectively (Supplementary Table 3, 4). One bidirectional, rho-independent transcription terminator was identified in each phage genome (Supplementary Fig. 2) and experimentally confirmed (unpublished result).

**Fig. 2:**
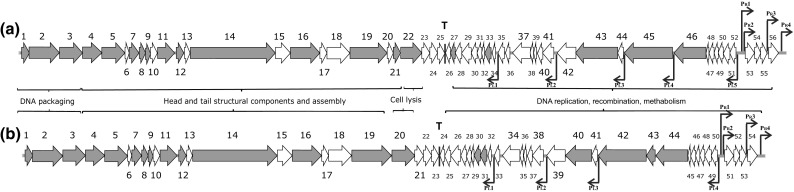
Genomic organization of *φ*673 (a) and *φ*674 (b) phages. ORFs are numbered consecutively from left to right and are indicated by arrows in the direction of transcription. ORFs encoding proteins assigned to known biological functions are depicted as dark arrows while those without known function are light. ORFs, joined by braces, are indicated to represent the proposed functional modules within the phage genomes. Promoter positions and directions are indicated by thin arrows, while intrinsic terminators are indicated by boxes

Based on homology to known phage proteins, functional domains, and mutual arrangement, putative functions were assigned to products of 20 of the predicted ORFs in each phage (Supplementary Table 1, 2). For each phage, the entire genome was divided into four functional modules (Fig. 2). The DNA packaging module includes small (gp_φ673_1 and gp_φ674_1) and large (gp_φ673_2 and gp_φ674_2) terminase subunits and a portal protein (gp_φ673_3 and gp_φ674_3). A head maturation protease (gp_φ673_4 and gp_φ674_4), major capsid and tail proteins (gp_φ673_5 and gp_φ674_5 and gp_φ673_11 and gp_φ674_11), head-to-tail connectors (gp_φ673_7, 8, 9 and gp_φ674_7, 8, 9), a tail assembly chaperone (gp_φ673_12 and gp_φ674_12), a tail TMP (gp_φ673_14 and gp_φ674_14), a tail protein (gp_φ673_16 and gp_φ674_16) and a tail fiber protein (gp_φ673_19, 21 and gp_φ674_19) could be predicted in the structural components and assembly module. Two major structural proteins for each virion, the major capsid (gp_φ673_5 and gp_φ674_5) and tail (gp_φ673_11 and gp_φ674_11) proteins, were detected via SDS-PAGE and identified via trypsin-based peptide mass fingerprinting (PMF) using an Ultraflex II LC-MALDI-TOF/TOF (Bruker) in accordance with a previously described procedure [10] (Fig. 1c, d, e). Furthermore, elimination of an N-terminal Met residue retained in trypsin-digested peptides from gp_φ673_11 and gp_φ674_11 confirmed the predicted N-terminal processing rule [11] (Fig. 1c, e).

A homolog of a known enzyme, lysozyme-like protein (gp_φ673_22 and gp_φ674_20), was predicted in the host lysis module. The replication/recombination/metabolism module also contained homologs to known proteins, including helicase (gp_φ673_43 and gp_φ674_40), the DNA replication protein RepA primase/helicase (gp_φ673_45 and gp_φ674_42), DNA polymerase I (gp_φ673_ 46 and gp_φ674_ 44) and HNH endonuclease (gp_φ673_33 and gp_φ674_30, 31, 43). One transcriptional regulator, gp_φ673_ 27, was identified. Interestingly, a putative intein was identified in the helicase encoded in ORF 43 for *φ*673, in contrast to the helicase encoded in ORF 40 for *φ*674, which exhibited no inteins. It has previously been reported that the *Corynebacterium* phage P1201 contains inteins [3].

Significant similarity throughout the genome was observed between the two newly sequenced and annotated lytic corynephages, *φ*673 and *φ*674, which exhibited approximately 85.2% identity. A bioinformatics search revealed that both phage genomes had high similarity to the genome of the corynephage BFK20 [2], with approximately 55% identity. Multiple genome alignment was constructed with Mauve (ver. 2.2.0) (Supplementary Fig. 3).

Besides *C. glutamicum* ATCC 13032, the host strain for both *φ*673 and *φ*674 phages, MB001 (prophage-free variant of *C. glu* ATCC 13032) was also infected by both phages. Another tested wild-type strain *Brevibacterium lactofermentum* AJ1511 was not lysed by either of the two phages.

We identified four *C. glutamicum* ATCC13032 genes, responsible for phage *φ*674 sensitivity (unpublished results). Two of these genes encoded glycosyltransferases; these proteins are bacterial sugar transferases involved in lipopolysaccharide synthesis. The third gene is annotated as a gene encoding a putative secreted protein. The fourth gene encodes a nucleotidyltransferase/DNA polymerase involved in DNA repair that is a DNA polymerase IV homolog. We hypothesized that these glycosyltransferases participate in the synthesis of a *φ*674 phage receptor containing an unknown sugar component in its structure [12].

In summary, the genomes of the *φ*673 and *φ*674 phages are significantly different from existing corynephage genomes available in databases; therefore, the sequences of these complete phage genomes were deposited for the first time in GenBank under accession numbers: MG324353, MG324354.

## Electronic supplementary material

Below is the link to the electronic supplementary material.

Supplementary material 1 (DOC 455 kb)
