# Supplementary material for: Complete nucleotide sequences and annotations of φ673 and φ674, two newly characterised lytic phages of Corynebacterium glutamicum ATCC 13032
Source: Arch Virol. 2018 May 15;163(9):2565–8. doi: 10.1007/s00705-018-3867-x (PMC6132909; doi:10.1007/s00705-018-3867-x)
Supplement: Supplementary file 1 — Supplementary material 1 (DOC 455 kb) [file 705_2018_3867_MOESM1_ESM.doc]

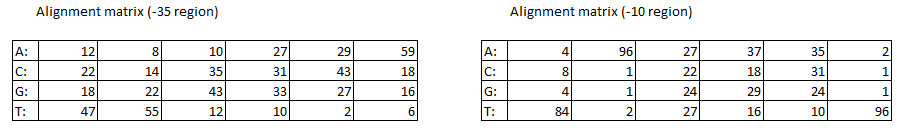


**Supplementary Fig. 1** The input alignment matrices for the phiSITE Promoter Hunter program for the “-10” and
“-35” motifs, calculated on the basis of data for *Corynebacterium glutamicum* housekeeping promoters, published by Pfeifer-Sancar et al.[[1]](#footnote-2)

**Supplementary Fig. 2**

**The bidirectional rho–independent transcription terminators of *φ*673 and *φ*674 phages.**

| Phage | Strands | Colored secondary structures: loops are in red  and stems in blue (underlined). | Free energy of stem-loop region (kcal/mol). |
| --- | --- | --- | --- |
| *φ*673 | Both +  🡪 | ATAAAAATAACGCCCCACCTTGATAATGAGGTGGGGCgTTATTTTTGTGT | -18.1 |
| *φ*673 | Both –  🡨 | ACAAAAATAACGCCCCACCTCATTATCAAGGTGGGGCgTTATTTTTATTC | -18.1 |
| *φ*674 | Both +  🡪 | ACAAAAATAACGCCCCACCTTGATAATGAGGTGGGGCgTTATTTTTGTGT | -18.1 |
| *φ*674 | Both –  🡨 | ACAAAAATAACGCCCCACCTCATTATCAAGGTGGGGCgTTATTTTTGTTA | -18.1 |

Rho-independent terminators were searched by “ARNold” program.

Hairpin structure for predicted terminators was analyzed by RNAstructure v5.8.1 (http://rna.urmc.rochester.edu/RNAstructure.html).

1. (b) (c) (d)


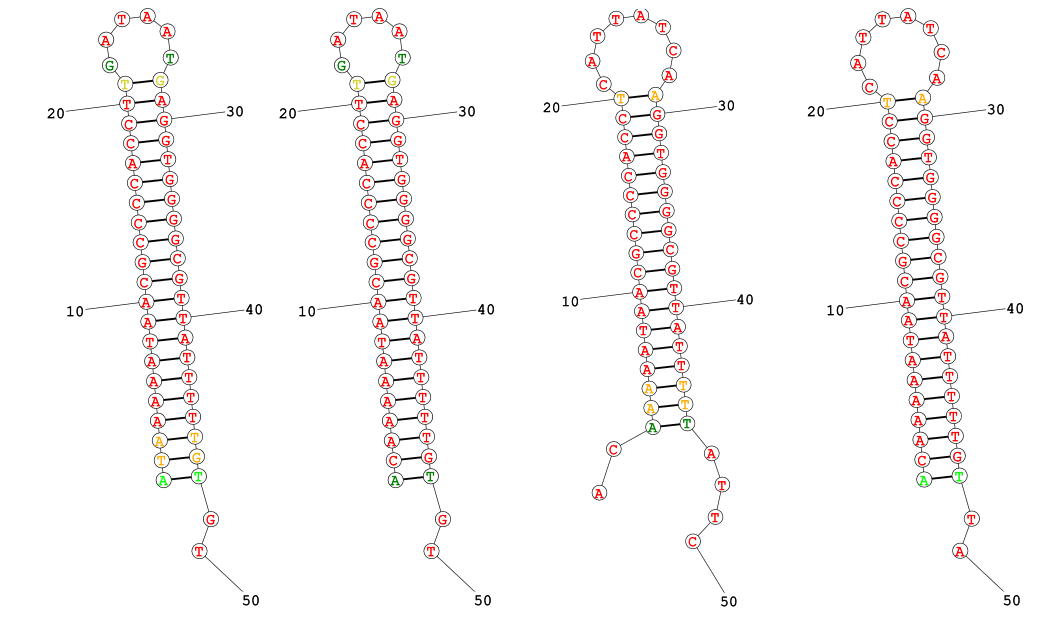


Result of ClustalW analysis of predicted terminators (a) *φ*673 direct; (b) *φ*674 direct; (c) *φ*673 complement; (d) φ674 complement.

**
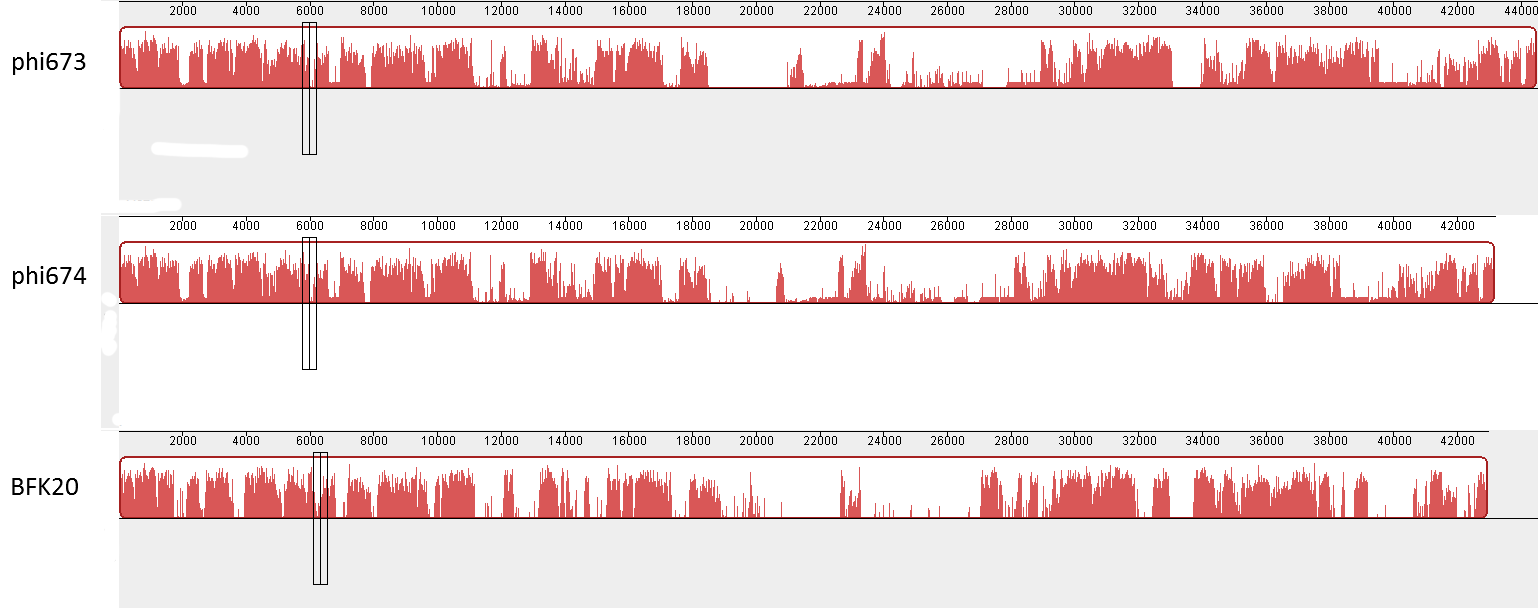
Supplementary Fig.3** Multiple genome alignment of *φ*673, *φ*674 genome sequences and related phage BFK20 sequence. Multiple genome alignment was constructed with Mauve (ver. 2.2.0). For analysis, the whole phage genome sequences of *φ*673, *φ*674 were used together with BFK20 phage sequence.

**Supplementary Table 1** *φ*673 ORFs, gene products, and functional assignments

| **ORF** | **Direction** | **START** | **STOP** | **Length** | **Predicted function** | **Related proteins** | **GeneBank accesion** | **E-value** | **Identity** |
| --- | --- | --- | --- | --- | --- | --- | --- | --- | --- |
| 1 | + | 126 | 563 | 438 | **Phage terminase,** | gp1 [Corynebacterium phage BFK20] | YP_001456731.1 | 2E-49 | 62% |
|  |  |  |  |  | **small subunit*** |  |  |  |  |
| 2 | + | 544 | 2226 | 1683 | **Phage terminase, large subunit (IPR005021)** | gp2, terminase [Corynebacterium phage BFK20] terminase [Corynebacterium aquilae] | YP_001456732.1 WP_084563006.1 | 0  7E-163 | 69%  49% |
| 3 | + | 2236 | 3516 | 1281 | **Portal protein**  **(IPR006944)** | gp3, phage portal protein [Corynebacterium phage BFK20] | YP_001456733.1 | 0 | 62% |
|  |  |  |  |  |  | portal protein [Gordonia phage Vendetta] | YP_009273941.1 | 1E-102 | 41% |
| 4 | + | 3518 | 4603 | 1086 | **Head maturation protease** | gp5, head maturation protease [Corynebacterium phage BFK20] | YP_001456735.1 | 2E-160 | 66% |
|  |  |  |  |  | **(IPR006433)** | HK97 family phage prohead protease [Corynebacterium diphtheriae] | WP_072574484.1 | 7E-120 | 54% |
| 5 | + | 4593 | 5876 | 1284 | **Major capsid protein (IPR006444)** | gp6, major capsid protein [Corynebacterium phage BFK20] | YP_001456736.1 | 6E-165 | 62% |
|  |  |  |  |  |  | phage major capsid protein [Corynebacterium aquilae] | WP_075727251.1 | 2E-139 | 53% |
| 6 | + | 5886 | 6116 | 231 |  | gp7, [Corynebacterium phage BFK20] | YP_001456737.1 | 8,E-12 | 52% |
| 7 | + | 6120 | 6665 | 546 | **Head-to-tail** | gp8 [Corynebacterium phage BFK20] | YP_001456738.1 | 7E-63 | 55% |
|  |  |  |  |  | **connector** | head-to-tail connector [Arthrobacter phage Galaxy] | ALY08855.1 | 0,0000003 | 26% |
| 8 | + | 6677 | 7033 | 357 | **Head-to-tail**  **connector** | gp9 [Corynebacterium phage BFK20]  head-to-tail connector protein [Gordonia phage Blueberry]  head-to-tail connector protein [Gordonia phage Vendetta] | YP_001456739.1  YP_009276634.1  YP_009273946.1 | 1E-41  1E-15  1E-15 | 60%  37%  40% |
| 9 | + | 7026 | 7310 | 285 | **Head-to-tail** | gp10 [Corynebacterium phage BFK20] | YP_001456740.1 | 7E-34 | 77% |
|  |  |  |  |  | **connector** | head-to-tail connector protein [Gordonia phage Vendetta] | YP_009273947.1 | 0,087 | 54% |
| 10 | + | 7307 | 7684 | 378 |  | gp11 [Corynebacterium phage BFK20] | YP_001456741.1 | 2E-41 | 63% |
| 11 | + | 7702 | 8649 | 948 | **Major tail** | gp12, major tail protein [Corynebacterium phage BFK20] | YP_001456742.1 | 9E-149 | 66% |
|  |  |  |  |  | **protein** | major tail protein [Gordonia phage Huffy] | AQY55621.1 | 1E-28 | 36% |
|  |  |  |  |  |  | major tail protein [Gordonia phage Vendetta] | YP_009273950.1 | 1E-28 | 36% |
| 12 | + | 8746 | 9159 | 414 | **Tail assembly** | gp13 [Corynebacterium phage BFK20] | YP_001456743.1 | 9E-34 | 48% |
|  |  |  |  |  | **chaperone** | tail assembly chaperone [Gordonia phage Ghobes] | YP_009281116.1 | 0,000009 | 28% |
| 13 | + | 9195 | 9509 | 315 |  | gp14 [Corynebacterium phage BFK20] | YP_001456744.1 | 2E-14 | 45% |
| 14 | + | 9525 | 14258 | 4734 | **Tape measure** | gp15, minor tail protein [Corynebacterium phage BFK20] | YP_001456745.1 | 0 | 58% |
|  |  |  |  |  | **protein (IPR010090)** | tape measure protein [Rhodococcus phage ReqiDocB7] | YP_009013812.1 | 2E-85 | 40% |
| 15 | + | 14270 | 15100 | 831 |  | gp16 [Corynebacterium phage BFK20] | YP_001456746.1 | 4E-57 | 40% |
| 16 | + | 15107 | 16735 | 1629 | **Tail protein** | Gp18, tail protein [Corynebacterium striatum] | CQD13435.1 | 0 | 59% |
|  |  |  |  |  |  | gp18, tail protein [Corynebacterium phage BFK20] | YP_001456748.1 | 7E-170 | 68% |
| 17 | + | 16748 | 17137 | 390 |  | gp19 [Corynebacterium phage BFK20] | YP_001456749.1 | 5E-40 | 55% |
| 18 | + | 17137 | 18411 | 1275 |  | gp20 [Corynebacterium phage BFK20] | YP_001456750.1 | 9E-136 | 53% |
|  |  |  |  |  |  | gp51 [Corynebacterium phage P1201] | YP_001468953.1 | 1E-24 | 25% |
| 19 | + | 18426 | 20495 | 2070 | **Tail fiber protein** | gp40 [Corynebacterium phage P1201] | YP_001468942.1 | 2E-178 | 67% |
|  |  |  |  |  |  | gp22 [Corynebacterium phage BFK20] | YP_001456752.1 | 2E-146 | 62% |
|  |  |  |  |  |  | gp21, tail fiber protein [Corynebacterium phage BFK20] | YP_001456751.1 | 3,E-26 | 42% |
| 20 | + | 20497 | 20835 | 339 |  | gp23 [Corynebacterium phage BFK20] | YP_001456753.1 | 4E-55 | 75% |
|  |  |  |  |  |  | gp41 [Corynebacterium phage P1201] | YP_001468943.1 | 5E-23 | 41% |
| 21 | + | 20832 | 21236 | 405 | **Tail fiber protein** | right-handed parallel beta-helix repeat-containing protein [Corynebacterium efficiens] | WP_011075173.1 | 2E-20 | 42% |
|  |  |  |  |  |  | gp21, tail fiber protein [Corynebacterium phage BFK20] | YP_001456751.1 | 1E-12 | 37% |
|  |  |  |  |  |  | tail fiber protein [Corynebacterium phage IME1320_01] | ARM68441.1 | 2E-09 | 34% |
| 22 | + | 21239 | 22519 | 1281 | **Lysozyme** | lysin A [Mycobacterium phage Milly] | YP_009125502.1 | 1E-58 | 45% |
|  |  |  |  |  | **(lysin A)** | hypothetical protein [Mycobacterium fortuitum] | WP_081392715.1 | 2E-58 | 43% |
|  |  |  |  |  |  | lysin A [Mycobacterium phage Marcoliusprime] | AOZ64367.1 | 2E-58 | 53% |
| 23 | + | 22512 | 23000 | 489 |  | hypothetical protein [Corynebacterium glutamicum] | WP_011897438.1 | 1E-17 | 41% |
|  |  |  |  |  |  | hypothetical protein [Corynebacterium falsenii] | WP_065420077.1 | 2E-12 | 44% |
| 24 | + | 22997 | 23461 | 465 |  | gp27 [Corynebacterium phage BFK20] | YP_001456757.1 | 1E-31 | 45% |
| 25 | + | 23461 | 23901 | 441 |  | gp28 [Corynebacterium phage BFK20] | YP_001456758.1 | 2E-18 | 36% |
| 26 | - | 23949 | 24245 | 297 |  | None identified |  |  |  |
| 27 | - | 24249 | 24527 | 279 | **Transcriptional regulator** | XRE family transcriptional regulator [Corynebacterium flavescens] | WP_075730389.1 | 6E-30 | 68% |
|  |  |  |  |  |  | MULTISPECIES: XRE family transcriptional regulator [Mycobacterium] | WP_090433947.1 | 3E-21 | 56% |
| 28 | - | 24529 | 24918 | 390 |  | None identified |  |  |  |
| 29 | - | 24911 | 25498 | 588 |  | None identified |  |  |  |
| 30 | - | 25500 | 25853 | 354 |  | None identified |  |  |  |
| 31 | - | 25850 | 26137 | 288 |  | None identified |  |  |  |
| 32 | - | 26134 | 26310 | 177 |  | None identified |  |  |  |
| 33 | - | 26307 | 26696 | 390 | **HNH** | gp29 [Corynebacterium phage BFK20] | YP_001456759.1 | 1E-48 | 67% |
|  |  |  |  |  | **endonuclease** | HNH endonuclease [Amycolatopsis kentuckyensis] | WP_086846745.1 | 0,0001 | 49% |
| 34 | - | 26765 | 26965 | 201 |  | None identified |  |  |  |
| 35 | - | 27115 | 27498 | 384 |  | None identified |  |  |  |
| 36 | - | 27567 | 27833 | 267 |  | None identified |  |  |  |
| 37 | - | 27991 | 29070 | 1080 |  | gp37 [Corynebacterium phage BFK20] | YP_001456767.1 | 3E-27 | 43% |
|  |  |  |  |  |  | DUF3310 domain-containing protein [Corynebacterium variabile] | WP_014009393.1 | 0,0000004 | 44% |
| 38 | - | 29052 | 29261 | 210 |  | None identified |  |  |  |
| 39 | - | 29258 | 29413 | 156 |  | None identified |  |  |  |
| 40 | - | 29474 | 29797 | 324 |  | None identified |  |  |  |
| 41 | - | 29800 | 30486 | 687 |  | gp39 [Corynebacterium phage BFK20] | YP_001456769.1 | 4E-30 | 48% |
| 42 | - | 30684 | 31820 | 1137 |  | gp40 [Corynebacterium phage BFK20] | YP_001456770.1 | 5E-160 | 66% |
|  |  |  |  |  |  | gp49 [Mycobacterium phage Akoma] | YP_009018559.1 | 7E-49 | 33% |
| 43 | - | 31833 | 34343 | 2511 | **Helicase** | gp41, helicase [Corynebacterium phage BFK20] | YP_001456771.1 | 0 | 72% |
|  |  |  |  |  |  | type III restriction protein [Mycobacterium phage Adawi] | YP_008530925.1 | 0% | 41% |
| 44 | - | 34340 | 34690 | 351 |  | None identified |  |  |  |
| 45 | - | 34744 | 37635 | 2892 | **RepA like protein** | gp43, RepA like protein [Corynebacterium phage BFK20] | YP_001456773.2 | 0 | 61% |
|  |  |  |  |  |  | DNA primase/helicase [Mycobacterium phage Vivaldi] | AIM50291.1 | 3E-87 | 36% |
| 46 | - | 37743 | 39677 | 1935 | **DNA polymerase I (IPR019760, IPR002562)** | gp44, DNA polymerase I [Corynebacterium phage BFK20]  DNA polymerase I [Mycobacterium phage Adawi] | YP_001456774.1 YP_008530933.1 | 0  4E-154 | 59%  47% |
| 47 | - | 39690 | 39863 | 174 |  | None identified |  |  |  |
| 48 | - | 39863 | 40168 | 306 |  | None identified |  |  |  |
| 49 | - | 40165 | 40419 | 255 |  | None identified |  |  |  |
| 50 | - | 40416 | 40721 | 306 |  | None identified |  |  |  |
| 51 | - | 40718 | 41119 | 402 |  | None identified |  |  |  |
| 52 | - | 41109 | 41486 | 378 |  | gp48 [Corynebacterium phage BFK20] | YP_001456778.1 | 5E-18 | 39% |
| 53 | + | 42171 | 42611 | 441 |  | None identified |  |  |  |
| 54 | + | 42618 | 42983 | 366 |  | gp50 [Corynebacterium phage BFK20] | YP_001456780.1 | 0,004 | 41% |
| 55 | + | 42976 | 43368 | 393 |  | gp52 [Corynebacterium phage BFK20] | YP_001456782.1 | 4E-61 | 72% |
| 56 | + | 43461 | 44057 | 597 |  | gp54 [Corynebacterium phage BFK20] | YP_001456784.1 | 1E-27 | 35% |

**Supplementary Table 2** *φ* 674 ORFs, gene products, and functional assignments

| **ORF** | **Direction** | **START** | **STOP** | **Length** | **Predicted function** | **Related proteins** | **GeneBank accesion** | **E-value** | **Identity** |
| --- | --- | --- | --- | --- | --- | --- | --- | --- | --- |
| 1 | + | 128 | 565 | 438 | **Phage terminase,** | gp1 [Corynebacterium phage BFK20] | YP_001456731.1 | 2,00E-49 | 61% |
|  |  |  |  |  | **small subunit*** |  |  |  |  |
| 2 | + | 546 | 2228 | 1683 | **Phage terminase,**  **large subunit (IPR005021)** | gp2, terminase [Corynebacterium phage BFK20]  terminase [Corynebacterium aquilae] | YP_001456732.1  WP_084563006.1 | 0  1,00E-163 | 69%  49% |
| 3 | + | 2238 | 3503 | 1266 | **Portal protein (IPR006944)** | gp3, phage portal protein [Corynebacterium phage BFK20]  portal protein [Gordonia phage Vendetta] | YP_001456733.1  YP_009273941.1 | 0  3,00E-102 | 63%  42% |
| 4 | + | 3505 | 4590 | 1086 | **Head maturation**  **protease (IPR006433)** | gp5, head maturation protease [Corynebacterium phage BFK20]  HK97 family phage prohead protease [Corynebacterium doosanense] | YP_001456735.1  WP_018021460.1 | 5,00E-159  6,00E-121 | 65%  52% |
| 5 | + | 4580 | 5860 | 1281 | **Major capsid**  **protein**  **(IPR006444)** | gp6, major capsid protein [Corynebacterium phage BFK20]  phage major capsid protein [Corynebacterium aquilae] | YP_001456736.1  WP_075727251.1 | 5,00E-166  9,00E-139 | 62%  52% |
| 6 | + | 5860 | 6093 | 234 |  | gp7, [Corynebacterium phage BFK20] | YP_001456737.1 | 1,00E-11 | 58% |
| 7 | + | 6097 | 6642 | 546 | **Head-to-tail**  **connector** | gp8 [Corynebacterium phage BFK20]  head-to-tail connector protein [Gordonia phage Eyre] | YP_001456738.1  YP_009292399.1 | 4,00E-62  8,00E-07 | 55%  34% |
|  |  |  |  |  |  | gp9 [Corynebacterium phage BFK20] | YP_001456739.1 | 4,00E-41 | 60% |
| 8 | + | 6654 | 7010 | 357 | **Head-to-tail**  **connector** | head-to-tail connector protein [Gordonia phage Blueberry] | YP_009276634.1 | 2,00E-15 | 37% |
|  |  |  |  |  |  | head-to-tail connector protein [Gordonia phage Vendetta] | YP_009273946.1 | 2,00E-15 | 41% |
|  |  |  |  |  |  | gp10 [Corynebacterium phage BFK20] | YP_001456740.1 | 7,00E-35 | 79% |
| 9 | + | 7003 | 7287 | 285 | **Head-to-tail**  **connector** | head-to-tail connector protein [Gordonia phage Ghobes] | YP_009281112.1 | 2,00E-04 | 36% |
|  |  |  |  |  |  | head-to-tail connector protein [Gordonia phage Vendetta] | YP_009273947.1 | 0,086 | 54% |
| 10 | + | 7284 | 7661 | 378 |  | gp11 [Corynebacterium phage BFK20] | YP_001456741.1 | 4,00E-40 | 60% |
| 11 | + | 7679 | 8626 | 948 | **Major tail protein**  **(IPR010090)** | gp12, major tail protein [Corynebacterium phage BFK20]  major tail protein [Gordonia phage Huffy]  major tail protein [Gordonia phage Vendetta] | YP_001456742.1  AQY55621.1 YP_009273950.1 | 4,00E-148  3,00E-29 3,00E-29 | 66%  36%  36% |
| 12 | + | 8720 | 9133 | 414 | **Tail assembly**  **chaperone** | gp13 [Corynebacterium phage BFK20]  tail assembly chaperone [Gordonia phage Ghobes] | YP_001456743.1  YP_009281116.1 | 2,00E-33  5,00E-06 | 47%  28% |
| 13 | + | 9169 | 9483 | 315 |  | gp14 [Corynebacterium phage BFK20] | YP_001456744.1 | 2,00E-14 | 45% |
| 14 | + | 9499 | 14217 | 4719 | **Tape measure**  **protein** | gp15, minor tail protein [Corynebacterium phage BFK20]  tape measure protein [Rhodococcus phage ReqiDocB7] | YP_001456745.1  YP_009013812.1 | 0  1,00E-84 | 58%  39% |
| 15 | + | 14229 | 15059 | 831 |  | gp16 [Corynebacterium phage BFK20] | YP_001456746.1 | 7,00E-58 | 40% |
| 16 | + | 15066 | 16694 | 1629 | **Tail protein** | gp18, tail protein [Corynebacterium striatum] | CQD13435.1 | 0 | 59% |
|  |  |  |  |  |  | gp18, tail protein [Corynebacterium phage BFK20] | YP_001456748.1 | 9,00E-171 | 68% |
| 17 | + | 16707 | 17096 | 390 |  | gp19 [Corynebacterium phage BFK20] | YP_001456749.1 | 3,00E-40 | 57% |
| 18 | + | 17096 | 18376 | 1281 |  | gp20 [Corynebacterium phage BFK20] | YP_001456750.1 | 2,00E-130 | 52% |
| 19 | + | 18390 | 20606 | 2217 | **Tail fiber protein (IPR011050)** | Pectate lyase superfamily protein [Pseudoxanthomonas sp. CF385]  cell wall-binding repeat protein [Lachnoanaerobaculum saburreum DSM 3986] | SDR04706.1  EFU77849.1 | 3,00E-40  4,00E-39 | 37%  40% |
|  |  |  |  |  |  | gp21, tail fiber protein [Corynebacterium phage BFK20] | YP_001456751.1 | 4,00E-16 | 32% |
| 20 | + | 20634 | 21920 | 1287 | **Lysozyme (lysin A)**  **(IPR023346,IPR016047)** | lysin A [Mycobacterium phage Milly]  lysin A [Mycobacterium phage Marcoliusprime] | YP_009125502.1  AOZ64367.1 | 8,00E-62  1,00E-61 | 47%  55% |
| 21 | + | 21910 | 22398 | 489 |  | hypothetical protein [Corynebacterium glutamicum]  hypothetical protein [Corynebacterium falsenii] | WP_011897438.1  WP_065420077.1 | 1,00E-17  8,00E-13 | 41%  45% |
| 22 | + | 22395 | 22859 | 465 |  | gp27 [Corynebacterium phage BFK20] | YP_001456757.1 | 2,00E-31 | 45% |
| 23 | + | 22859 | 23296 | 438 |  | gp28 [Corynebacterium phage BFK20] | YP_001456758.1 | 5,00E-20 | 36% |
| 24 | - | 23347 | 23643 | 297 |  | None identified |  |  |  |
| 25 | - | 23640 | 24005 | 366 |  | None identified |  |  |  |
| 26 | - | 23998 | 24618 | 621 |  | None identified |  |  |  |
| 27 | - | 24620 | 24973 | 354 |  | None identified |  |  |  |
| 28 | - | 24970 | 25254 | 285 |  | None identified |  |  |  |
| 29 | - | 25251 | 25430 | 180 |  | None identified |  |  |  |
| 30 | - | 25427 | 25816 | 390 | **HNH endonuclease** | gp29 [Corynebacterium phage BFK20]  HNH endonuclease [Amycolatopsis kentuckyensis] | YP_001456759.1  WP_086846745.1 | 2,00E-50  2,00E-04 | 69%  49% |
| 31 | - | 25806 | 26210 | 405 | **HNH**  **endonuclease** | gp29 [Corynebacterium phage BFK20]  HNH endonuclease [Streptomyces mangrovisoli] | YP_001456759.1 WP_046591576.1 | 3,00E-191,00E-05 | 45%  41% |
| 32 | - | 26286 | 26486 | 201 |  | None identified |  |  |  |
| 33 | - | 26665 | 27003 | 339 |  | None identified |  |  |  |
| 34 | - | 27162 | 28226 | 1065 |  | gp37 [Corynebacterium phage BFK20]  DUF3310 domain-containing protein [Corynebacterium variabile] | YP_001456767.1  WP_014009393.1 | 3,00E-27  3,00E-07 | 43%  44% |
| 35 | - | 28223 | 28432 | 210 |  | None identified |  |  |  |
| 36 | - | 28429 | 28584 | 156 |  | None identified |  |  |  |
| 37 | - | 28691 | 28984 | 294 |  | None identified |  |  |  |
| 38 | - | 28987 | 29661 | 675 |  | gp39 [Corynebacterium phage BFK20] | YP_001456769.1 | 2,00E-31 | 49% |
| 39 | - | 29859 | 30995 | 1137 |  | gp40 [Corynebacterium phage BFK20] | YP_001456770.1 | 3,00E-160 | 66% |
| 40 | - | 31008 | 32612 | 1605 | **Helicase**  **(IPR014001)** | gp49 [Mycobacterium phage Akoma]gp41, helicase [Corynebacterium phage BFK20]  helicase [Tsukamurella phage TPA2] | YP_009018559.1  YP_001456771.1  YP_004286299.1 | 6,00E-49  0  3,00E-133 | 33%  72%  45% |
| 41 | - | 32609 | 32959 | 351 |  | None identified |  |  |  |
| 42 | - | 33013 | 35931 | 2919 | **RepA like protein**  **(IPR015330)** | gp43, RepA like protein [Corynebacterium phage BFK20]  gp57 [Mycobacterium phage UncleHowie] | YP_001456773.2  YP_009168237.1 | 0  9,00E-86 | 59%  35% |
| 43 | - | 35948 | 36478 | 531 | **HNH endonuclease**  **(IPR003615)** | HNH endonuclease [Devosia epidermidihirudinis] | WP_082092497.1 | 4,00E-34 | 41% |
| 44 | - | 36535 | 38463 | 1929 | **DNA polymerase I**  (**IPR001098)** | gp44, DNA polymerase I [Corynebacterium phage BFK20]  gp54 [Mycobacterium phage Cooper] | YP_001456774.1  YP_654951.1 | 0  4,00E-153 | 61%  45% |
| 45 | - | 38476 | 38649 | 174 |  | None identified |  |  |  |
| 46 | - | 38649 | 38954 | 306 |  | None identified |  |  |  |
| 47 | - | 38951 | 39205 | 255 |  | None identified |  |  |  |
| 48 | - | 39202 | 39507 | 306 |  | None identified |  |  |  |
| 49 | - | 39504 | 39905 | 402 |  | None identified |  |  |  |
| 50 | - | 39895 | 40272 | 378 |  | gp48 [Corynebacterium phage BFK20] | YP_001456778.1 | 4,00E-17 | 38% |
| 51 | + | 40834 | 41274 | 441 |  | None identified |  |  |  |
| 52 | + | 41281 | 41646 | 366 |  | gp50 [Corynebacterium phage BFK20] | YP_001456780.1 | 0,004 | 41% |
| 53 | + | 41639 | 42031 | 393 |  | gp52 [Corynebacterium phage BFK20] | YP_001456782.1 | 3,00E-61 | 72% |
| 54 | + | 42124 | 42720 | 597 |  | gp54 [Corynebacterium phage BFK20] | YP_001456784.1 | 3,00E-27 | 35% |

**Supplementary Table 3** Phage *φ* 673 putative promoters. Nucleotide sequences of predicted putative promoter “-10” and “-35” sequences are highlighted.

| Promoter | 5` -35 -10 3` |
| --- | --- |
| P_L1_ | TGGTTTAGGGGTTGCATGACCACATGTACGTGTGTACAGTATTAAGTAGA |
| P_L2_ | CATCCTCTGTGTGCAATGTCCTGGGTAGGACAT-TAAACTGCCCCCTGTA |
| P_L3_ | AGCGCAGACGTTGCGAGATGGGATCCAGTCCTAGTACAATCTATATTGCG |
| P_L4_ | ACATGGCTATTGGACAAATGGTTATGTAACCACCTATTCTAGATTGATAC |
| P_L5_ | CAATATGGGGTTGAAATCAGAGCATTAATGGTT-TAAAGTTATCCGTACC |
| P_R1_ | GGTATATAAGTTGACTCCATTTTTTTGGGTATGATAAGCTGTATCCACCG |
| P_R2_ | AGGTAGAAATTTCACCCTAAAAGTTGACTAA---CATAATTAAATGTGAT |
| P_R3_ | TAAGAACAGATTGAAATATTAACAACATGTAGTGTAATGTCTTAGTTGTG |
| P_R4_ | GATTAACTATTGACCAATGCGTTGCATATGTGTATATACTTGCTAGTGCA |

**Supplementary Table 4** Phage *φ* 674 putative promoters. Nucleotide sequences of predicted putative promoter “-10” and “-35” sequences are highlighted.

| Promoter | 5` -35 -10 3` |
| --- | --- |
| P_L1_ | TGGTTTAGGGGTTGCATGACCACATGCACATGTGTACAGTATTAAGTAGA |
| P_L2_ | CATCCTCTGTGTGCAATGTCCTGGGTAGGACAT-TAAACTGCCCCCTGTA |
| P_L3_ | AGCGCAGACGTTGCGAGATAGGATCCAGTCCTAGTACAATCTATATTGCG |
| P_L4_ | AAGTTATCCATACCGAGCAAAACACGCCGGT---TAGGGTGCACTTACTT |
| P_R1_ | GGTATATAAGTTGACTCCATTTTTTTGCATATGATAAGCTGTATCCACCG |
| P_R2_ | AGGTAGAAATTTCACCCTAAAAGTTGACTCA---CATAATTAAGTGTGAT |
| P_R3_ | TAAGAACAGATTGAAATATTAACAACATGTAGTGTAATGTCTTAGTTGTG |
| P_R4_ | GATTAACTATTGACCAATGCGTTGCACTTGTGTATATACTTGCAAGTGCA |

1. Pfeifer-Sancar K, Mentz A, Ruckert C, Kalinowski J (2013) Comprehensive analysis of the Corynebacterium glutamicum transcriptome using an improved RNAseq technique. BMC Genomics 14: 888. doi: 10.1186/1471-2164-14-888 [↑](#footnote-ref-2)
